# Variable alterations of the microbiota, without metabolic or immunological change, following faecal microbiota transplantation in patients with chronic pouchitis

**DOI:** 10.1038/srep12955

**Published:** 2015-08-12

**Authors:** J. Landy, A. W. Walker, J. V. Li, H. O. Al-Hassi, E. Ronde, N. R. English, E. R. Mann, D. Bernardo, S. D. McLaughlin, J. Parkhill, P. J. Ciclitira, S. K. Clark, S. C. Knight, A. L. Hart

**Affiliations:** 1 IBD Unit, Gastroenterology Dept. St Mark’s Hospital, Harrow, London, UK; 2 Antigen Presentation Research Group, Faculty of Medicine, Imperial College London, Northwick Park and St Mark’s Campus, Harrow, UK; 3 Pathogen Genomics Group, Wellcome Trust Sanger Institute, Hinxton, Cambridgeshire, UK; 4 Microbiology Group, Rowett Institute of Nutrition and Health, University of Aberdeen, Greenburn Road, Aberdeen, UK; 5 Centre for Digestive and Gut Health & Division of Computational and Systems Medicine, Department of Surgery and Cancer, Faculty of Medicine, Imperial College London, London, UK; 6 School of Health and Social Care, Bournemouth University, Bournemouth, UK; 7 Department of Gastroenterology, The Rayne Institute, St Thomas’ Hospital, London, UK; 8 Department of Surgery, St Mark’s Hospital, Harrow, London, UK

## Abstract

Faecal microbiota transplantation (FMT) is effective in the treatment of *Clostridium difficile* infection, where efficacy correlates with changes in microbiota diversity and composition. The effects of FMT on recipient microbiota in inflammatory bowel diseases (IBD) remain unclear. We assessed the effects of FMT on microbiota composition and function, mucosal immune response, and clinical outcome in patients with chronic pouchitis. Eight patients with chronic pouchitis (current PDAI ≥7) were treated with FMT via nasogastric administration. Clinical activity was assessed before and four weeks following FMT. Faecal coliform antibiotic sensitivities were analysed, and changes in pouch faecal and mucosal microbiota assessed by 16S rRNA gene pyrosequencing and ^1^H NMR spectroscopy. Lamina propria dendritic cell phenotype and cytokine profiles were assessed by flow cytometric analysis and multiplex assay. Following FMT, there were variable shifts in faecal and mucosal microbiota composition and, in some patients, changes in proportional abundance of species suggestive of a “healthier” pouch microbiota. However, there were no significant FMT-induced metabolic or immunological changes, or beneficial clinical response. Given the lack of clinical response following FMT via a single nasogastric administration our results suggest that FMT/bacteriotherapy for pouchitis patients requires further optimisation.

Inflammatory bowel diseases (IBD) occur as the result of an aberrant inter-relationship between the intestinal microbiota and host immune system in genetically predisposed individuals^1^,^2^. Modification of the microbiota is an emerging option for therapeutic purposes^3^. Some of the most compelling evidence for the clinical efficacy of altering the microbiota in IBD is derived from studies of pouchitis^4^, where intestinal inflammation occurs in the ileal reservoir after a restorative proctocolectomy (RPC) for ulcerative colitis (UC).

Microbiological data implicate a dysbiosis (i.e. an alteration in the composition of the microbiota to one that is deleterious to host health) in the pathogenesis of pouchitis, although a unifying or characteristic dysbiosis has not been demonstrated^5^,^6^,^7^. Clinical data confirm a role for antibiotics and probiotics for the treatment of acute and chronic pouchitis^8^,^9^,^10^,^11^,^12^,^13^,^14^. However, a proportion of patients either does not respond to antibiotic therapy, or become antibiotic dependent. Long term treatment with antibiotics may lead to the development of antibiotic resistance and promotion of extended spectrum beta-lactamase-producing (ESBL) bacteria^15^. Clinical studies report limited benefit for maintenance of remission with the probiotic combination, VSL#3^16^,^17^.

An alternative approach to conventional probiotics is transplantation of the entire “organ” of the gut microbiota from a healthy donor. Faecal microbiota transplantation (FMT) is effective in the treatment of recurrent *Clostridium difficile* infection, where resolution of disease is tightly linked to post-FMT changes in microbiota diversity and composition^18^. The effects of FMT on the recipient microbiota in *Clostridium difficile* and other gastrointestinal as well as metabolic conditions are durable^19^,^20^. Case reports and small case series have suggested positive clinical outcomes following FMT in IBD^20^,^21^,^22^,^23^. However, recent studies of FMT in UC did not demonstrate clinical efficacy and suggest variable efficiency of colonisation by the donors’ microbiota^24^,^25^. More recently, two randomised studies of FMT in active UC demonstrated conflicting clinical outcomes^26^,^27^. The ability of FMT to colonise the recipient microbial environment in the context of chronic inflammation remains unclear. To our knowledge, no studies have assessed the functional metabolic and immunological effects of FMT in the context of IBD.

Lamina propria dendritic cells (DC) are pivotal in the interactions between the host immune system and the gut microbiota due to their innate bacterial sensing properties and antigen sampling capacity^28^. Altered DC phenotype and function in IBD suggests that they contribute to inflammatory processes in response to the microbiota^28^,^29^. Probiotics and prebiotics have been shown to alter mucosal innate and adaptive immune responses, particularly DC function, in IBD^30^,^31^,^32^.

We hypothesised that FMT would induce clinical response in patients with chronic pouchitis, altering the composition and function of the pouch microbiota and elicit immunoregulatory effects. We aimed to assess the effect of FMT on the microbiota composition and function, in addition to effects on the mucosal immune response, particularly on DC, and assess the clinical response in patients with chronic pouchitis.

## Results

### Patient characteristics

Ten patients were recruited to the study. One withdrew prior to FMT. Another patient was diagnosed with adenocarcinoma of the rectal cuff at the screening pouchoscopy and did not proceed to FMT. Of the eight remaining patients studied, five were female. The median age was 46 years (range 24–63 years). The median number of years since restorative proctocolectomy (RPC) was 10 years (range 4–22 years) (Table 1).

### Alterations in antibiotic sensitivity post FMT

Ciprofloxacin resistant coliforms were detected in faecal samples from four patients. In three patients stool testing detected ESBL-coliforms (Table 1). In two patients who tested positive for ciprofloxacin-resistant coliforms in their stool samples prior to FMT (patients 3 and 5), coliforms regained sensitivity to ciprofloxacin post FMT. For these patients, regaining sensitivity to ciprofloxacin enabled further effective antimicrobial therapy during their on-going disease management.

### Clinical efficacy

There were no major adverse events following FMT. Three patients reported relatively minor adverse effects including nausea (n = 3); vomiting (n = 1); bloating (n = 2); fever (n = 1). All adverse events were transient, lasting less than 24 hours. However, following FMT, no patients achieved clinical remission. Two patients (patients 2 and 3) demonstrated a reduction of total PDAI score by ≥3 points at 4 weeks post FMT. However, both patients still had a total PDAI score ≥7 at 4 weeks post FMT. There were no changes in Cleveland global quality of life (QoL) score (Supplementary Figure 1).

### Microbiota analysis

**Figure 1:**
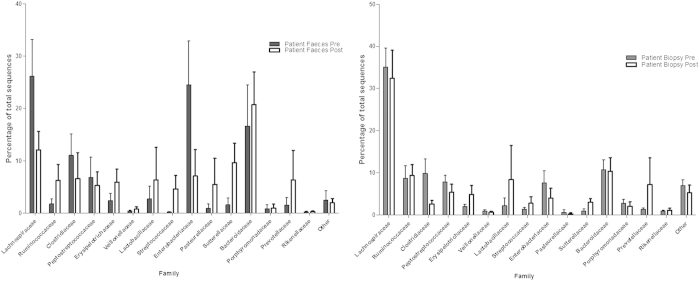
Analysis of bacterial families in stool and biopsy samples pre and post FMT. Percentage of sequences identified from the bacterial families of >1% total relative abundance in: (**A**) patient stool samples pre (n = 7) and post FMT (n = 8); (**B**) patient mucosal samples pre (n = 8) and post FMT (n = 5).

A total of 156,963 sequences were generated, with an average of 3982 reads per sample (range 391–12,592). Just over 1,000 operational taxonomic units (OTUs) were detected, with most sequences derived from the bacterial phyla *Firmicutes* (57%), *Bacteroidetes* (26%), *Proteobacteria* (14.5%) and *Actinobacteria* (1.6%) (Supplementary Figure 2). A total of 105 bacterial families were detected in samples, and 15 of these accounted for more than 90% of the total OTUs in the whole dataset (Fig. 1, Supplementary Figure 3).

### Donor and patient samples at baseline

There was significant inter-individual variation in microbiota content, in both the donors and pouch patients. Despite this, some general trends were observed. At the Phylum level, prior to FMT, pouchitis patient stool samples were characterised by a higher proportion of *Proteobacteria* (p = 0.0047) compared with donor stool samples (Supplementary Figure 2). At the Family level, patient stool samples prior to FMT were characterised by a lower proportion of common obligate anaerobic lineages such as *Ruminococcaceae* (p = <0.0001), *Coriobacteriaceae* (p = <0.0001), *Porphyromonadaceae* (p = 0.0003) and *Rikenellaceae* (p = 0.0007), and higher proportional abundances of *Enterobacteriaceae* (p = 0.004) and *Clostridiaceae* (p = 0.0097). (Supplementary Figure 3). Finally, when looking at the Genus and 97% operational taxonomic unit (OTU) levels, patient stool samples prior to FMT contained lower proportional abundances of many obligate anaerobes, including *Faecalibacterium prausnitzii* (p = 0.0002), and higher proportions of *Escherichia*/*Shigella* spp. (p = 0.0019) and *Ruminococcus gnavus* (p = 0.0029) compared with donor stool samples. (Supplementary Table 1).

Patient stool prior to FMT was characterised by low bacterial richness (observed OTUs p = 0.001) and diversity (Shannon diversity index p = 0.001; inverse Simpson diversity index p = 0.02; Chao 1 estimate of overall diversity p = 0.0003) compared with donor stool samples (Supplementary Figure 4).

### Changes in microbiota composition post faecal transplantation

**Figure 2:**
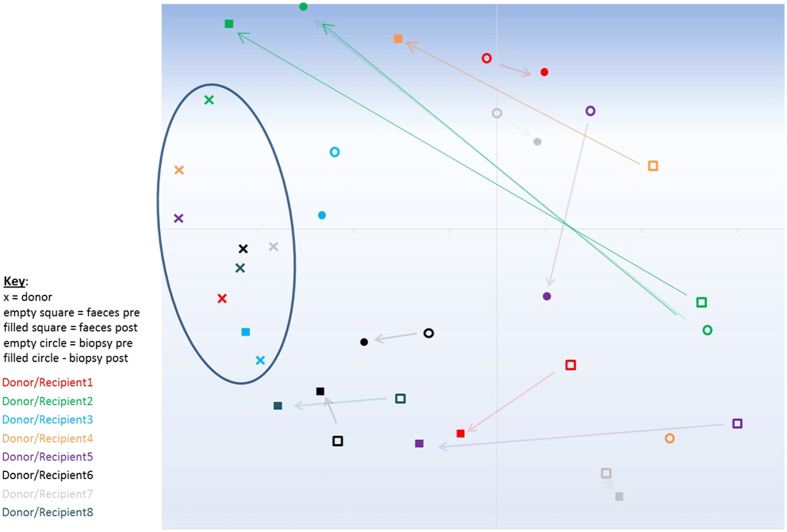
Non-metric multidimensional scaling (NMDS) analysis for donor and patient samples pre and post FMT. NMDS analysis, calculated in mothur using the Bray Curtis calculator, of donor stool (x) and patient stool pre FMT (open squares) and post FMT (filled squares) and patient mucosal samples pre FMT (open circles) and post FMT (filled circles) for each patient and donor. Oval shows distinct clustering of healthy donor faecal microbiota sample profiles in comparison to the pouchitis patient samples, arrows indicate directional shifts in patient samples post FMT.

There were no overall changes in bacterial richness or diversity of the faecal or mucosal microbiota post FMT (Supplementary Figure 4). However, Non-metric multidimensional scaling (NMDS) analysis using the Bray Curtis calculator, which measures dissimilarity in microbial community structure between samples by comparing overlapping OTU membership and their relative abundances, indicated a shift in both the stool and mucosal microbiota towards a composition with greater similarity to donor stool following FMT (Fig. 2). AMOVA analysis using the Yue and Clayton calculator, which also takes into account OTU membership and relative abundances when comparing bacterial community structures also suggested less difference between recipient and donor microbiota compositions following FMT (Donor vs recipient faeces pre FMT p = 0.005; Donor vs recipient faeces post FMT p = 0.034; Donor vs recipient biopsy pre FMT p = 0.003; Donor vs recipient biopsy post FMT p = 0.174).

The significant inter-individual variation of the baseline faecal and mucosal microbiota was also reflected in the varying responses following FMT by each of the recipient patients (Supplementary Figures 5). Broadly, Patients 2 and 3 showed a shift in their faecal and mucosal microbiota towards that of their respective donor’s stool following FMT. Patient 5 showed a shift in the faecal and mucosal microbiota post FMT, although this shift was not towards a similar microbiota to Donor 5. Patients 1 and 4 showed a slight shift in the composition of their faecal microbiota, but not of their mucosal microbiota. Patients 6, 7 and 8 showed no shift in either stool or mucosal microbiota following FMT. There was an increased proportion of *Ruminococcaceae* with a concomitant reduction in the proportion of *Enterobacteriaceae* in the samples post FMT in patients 2, 3, 4 and 5 (Supplementary Figure 5).

When averaging across the whole study cohort, *Escherichia coli/Shigella* spp. (p = 0.0051) and *Ruminococcus gnavus* (p = 0.0097) were reduced in proportional abundance in stool samples from patients post FMT, and there was an increase in relative abundance of *Sutterella stercoricanis* (p = 0.0024) and *Dorea longicatena* (p = 0.014) as well as a trend towards increased proportional abundance of *Faecalibacterium prauznitzii* (p = 0.0897). However, inter-individual variation was marked, and adjusting significance threshold levels to allow for false discovery rate using the Benjamini-Hochberg method suggested that none of these observations were significant. In biopsy samples the only significant difference at all taxonomic levels following FMT was a reduction in the proportional abundance of *Enterococcus* spp. (p = 0.001) but this group was not dominant in the mucosal microbiota pre-FMT (mean of 0.1% proportional abundance), and the p value only just reached the Benjamini-Hochberg corrected significance threshold. (Fig. 1) (Supplementary Table 2).

### ^1^H NMR spectroscopy-based metabonomic analysis of faecal samples from donors and patients

**Figure 3:**
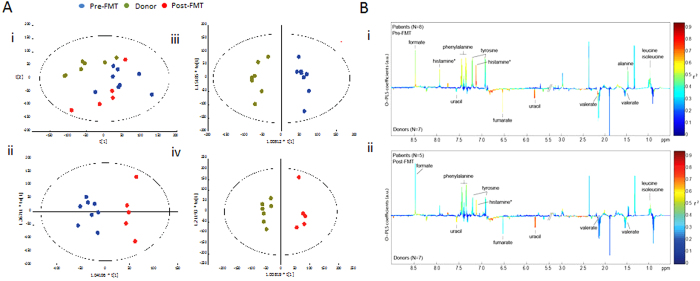
^1^H NMR-based metabonomic analysis of faecal samples from donors and patients. (**A**) PCA scores plot of ^1^H NMR profiles of fresh faecal water samples obtained from donors and patients at pre and post FMT (i). OPLS-DA scores plots of pre- and post FMT (ii, Q^2^Y < 0, p > 0.05), donors and patients at pre-FMT (iii, Q^2^Y = 0.81, p = 0.001), and donors and patients at post-FMT (iv, Q^2^Y = 0.72, p = 0.04). (**B**) O-PLS-DA loadings plots of ^1^H NMR profiles of fresh faecal water samples from donors and patients at pre-FMT (i) and donors and patients at post-FMT (ii). Peaks pointing upwards represent higher levels of metabolites in patients compared with donors and *vice versa*. The colours of peaks represent the correlation (r^2^) between the metabolites and the classification (e.g. patients or donors).

Results generated from fresh faecal water and dried faecal samples were similar. Strong metabolic differences were found between donors and patients after multiple test correction (Fig. 3). Higher levels of formate (p = 0.02), phenylalanine (p = 0.01), tyrosine (p = 0.02), leucine (p = 0.02), alanine (p = 0.02) and histamine (p = 0.002) (putatively assigned based on database correlation) were found in the patients in contrast to donors, whereas concentrations of uracil (p = 0.0006), fumarate (p = 0.01) and valerate (p = 0.006) were higher in donors (Fig. 3B) with a trend towards a higher level of acetate and butyrate in donors compared with patients. The variation in the metabolic data was demonstrated in the PCA scores plot, where samples sharing similar metabolic features cluster together. In the PCA scores plots (Fig. 3A), patients both pre- and post-FMT exhibited greater heterogeneity than donors. However, no significant metabolic changes in patients pre- and post-FMT (Fig. 3A ii) was observed as reflected by the negative values of Q^2^Y, a parameter describing the goodness of prediction.

Metabolic profiles of faecal water reveal the metabolites derived from the gut bacteria and the interactions between the host and bacteria. Therefore, we investigated the host-microbial crosstalk by integrating ^1^H NMR data with microbial profiles using O-PLS regression analysis. Of 17 selected bacterial groups, *Porphyromonadaceae* and unclassified *Firmicutes* levels showed positive correlative relationships with higher concentration of short chain fatty acids, uracil, fumarate and reverse correlation with lactate, succinate and histamine (Q^2^Y values of OPLS models are 0.52 and 0.43, respectively).

### Innate immune characteristics following FMT

There were also no significant changes after FMT in dendritic cell expression of TLR 2 (p = 0.3), TLR 4 (p = 0.2) or TLR 5 (p = 0.6). There were no significant changes in dendritic cell expression of homing markers β7 (p = 0.4) or CCR 9 (p = 0.8) before and after FMT or expression of the activation marker CD40 (p = 0.3) (Supplementary Figure 6). Furthermore, there was no significant reduction in the level of IL-6 (p = 0.5) or TNFα (p = 0.3) in the biopsy supernatants after FMT.

## Discussion

We report the first study of FMT in patients with chronic pouchitis that incorporates clinical, immunological, and both taxonomic and functional microbiological assessments. Our study suggests that administration of a single nasogastrically delivered FMT from a healthy donor results in some shift in the composition of the microbiota, and in some cases with specific changes in the abundance of species suggestive of a “healthier” pouch microbiota. However, microbiota engraftment success varied greatly between recipients. Regardless of engraftment success, FMT did not appear to result in significant functional change of the microbiota, mucosal immunological response or clinical efficacy.

Patients’ faecal microbiota differed significantly from healthy donors’ faecal microbiota, including reduced proportional abundances of *Faecalibacterium prausnitzii*, which has repeatedly been shown to be reduced in IBD patients and may have some anti-inflammatory properties^33^. This may be due to differences between the colonic and the pouch environment as well as disease specific differences. While many features of this dysbiosis may be driven by disease it is also possible that some may have resulted from prior antibiotic use. For practical and ethical reasons, we were only able to impose an antibiotic exclusion period of two weeks prior to FMT, and it has been shown previously that post-antibiotic microbiota perturbations can remain for longer periods than this^34^. However, ileal pouch faecal microbiota shifts towards a “colon-like” community following ileostomy closure^35^ and baseline pouch samples demonstrated findings in keeping with previous reports of dysbiosis in IBD and specifically pouchitis^5^,^6^,^7^,^24^,^25^,^36^.

Numerous studies document the association between increased *Proteobacteria*, specifically *Escherichia*, in IBD^37^,^38^,^39^. Post FMT, *Escherichia* were less proportionally abundant in a number of patients’ stools. Despite not reaching statistical significance, likely due to the large degree of inter-individual variation in microbiota content, other post FMT microbiota changes also suggested a slight overall shift towards a healthier bacterial composition. The mucolytic bacterium *Ruminococcus gnavus*, for example, has been previously shown to be increased in the epithelium of UC and CD patients^40^ and *Clostridiaceae* are associated with pouch inflammation (37). Furthermore, potentially beneficial bacterial groups were increased in proportional abundance in some individuals following FMT. For example, *Faecalibacterium prauznitzii* is reduced in colitis patients^41^ and has anti-inflammatory properties, *Dorea* has been associated with non-inflammatory pouch outcomes^7^ and a recent study associated increased abundance of *Sutterella* with pouch health^7^. It is of interest therefore that, despite these potentially beneficial shifts, no significant improvement in clinical outcome was detected.

Faecal metabolic profiles of patients with pouchitis were also similar to those of IBD patients reported previously^42^ and were characterized by higher levels of amino acids (e.g. alanine, isoleucine, leucine) and lower levels of short chain fatty acids in patients compared with non-IBD controls^43^. *Porphyromonadaceae* were positively correlated with elevated short chain fatty acid production, fumarate and uracil and negatively correlated with histamine, succinate and lactate. The higher abundance of *Porphyromonadaceae* in the donors or in healthy gut is in agreement with previous studies^44^. However, we did not observe any significant increase in *Porphyromonadaceae* post FMT and no significant metabolic changes in patients pre- and post-FMT were identified despite changes in the microbiota in some patients.

Abundant molecular functions are not necessarily provided by abundant species^45^ and core metabolic functions may be shared between bacteria, promoting stability in metabolic function, and maintaining homeostasis. Consequently, the changes in the microbiota that were demonstrated post FMT might not lead to significant metabolic and subsequently immunological changes.

The diversity of the pouch microbiota was reduced in the faecal samples, but not in the mucosal samples at baseline. This is an unexpected finding, but may represent the severity and longevity of inflammation in the mucosa of the patients included. Chronic inflammation may enable the residual mucosal microbiota to create a niche with reasonable diversity, whilst the high pouch frequency in these patients could decrease the stability of the faecal/luminal microbiota. Previous studies suggest a higher richness of the mucosal microbiota than the faecal microbiota with a greater impact on the diversity of the faecal compared with mucosal microbiota as a consequence of osmotic diarrhoea^46^. Higher species richness (“biodiversity”), enhances the robustness and stability of an ecosystem and might be an intrinsic safeguard against perturbations^47^.

The pouch frequency, and longevity/severity of inflammation in our patient cohort may explain the poorer outcomes in this study compared with a recent randomised study of FMT in UC patients^26^. Patients with milder or quiescent disease may therefore be more amenable to durable alteration of the microbiota with FMT. In the recent randomised studies of FMT in UC, the faecal microbiota following FMT showed greater diversity and there was a shift in responders towards a “healthier” composition of the microbiota. Whilst baseline Mayo score did not impact on treatment success, disease duration ≤1 year did impact on treatment success^26^. However, in another recent randomised study of FMT in patients with mild to moderate disease there was no significant clinical impact of FMT^27^.

There are also a number of possible methodological explanations for the lack of efficacy of FMT in this study compared with other recent studies. FMT was performed via a nasogastric tube in accordance with previously reported protocols for recurrent *Clostridium difficile* infection^48^,^49^ and indeed, the recently reported randomised controlled trial of FMT for recurrent *Clostridium difficile* employed nasoduodenal administration where benefit was demonstrated^18^. However, nasogastric tube delivery may not be the optimal mode of delivery for IBD patients. Colonic administration of donor stool was efficacious in reports of FMT for IBD^50^ and this may be a preferred and improved route of delivery for IBD patients. In the recent randomised studies of FMT for UC, where faecal enemas were used there was a significant difference in the proportion of patients in clinical remission compared with placebo^26^; whilst in the study using nasojejunal administration there was no significant difference between FMT from healthy donors and autologous FMT^27^. Previous reports of FMT for IBD also report the use of more frequent infusions, which may be necessary for more robust engraftment of donor microbiota. It is also unknown whether pre-treatment with bowel lavage and antibiotics is necessary, although in an animal model of FMT, antibiotics were not necessary to establish engraftment of exogenous microbiota^51^.

It is also possible that the microbiota from the donors used was suboptimal. Several of the donors were relatives or household contacts of the recipients. Relatives of patients with IBD have been shown to have alterations in their microbiota distinguishing them from other “healthy” donors^52^. In the two recent randomised studies of FMT in UC the majority of donors were unrelated^26^,^27^. The study by Moayyedi *et al*. also demonstrated differences in response depending on the donor used, with the majority of responders having received FMT from the two donors with similar microbiota profiles^26^. A study of FMT in mice showed that successful alteration of the recipient’s microbiota depended on the donor microbiota being both phylogenetically diverse and distinct from that of the recipient^53^. Here, donors were not selected in this way. Furthermore, the donor microbiota used here may not be applicable to the niche of the UC pouch and perhaps UC or familial adenomatous polyposis ileal pouch patients ***without*** a history of pouchitis would be a more appropriate donor in this context. However, no data are currently available regarding the optimal route of administration, volume or frequency of faecal infusion, adjunctive therapies or optimal properties of donor stool for either *Clostridium difficile* or IBD.

We conclude that a single FMT from a healthy donor by nasogastric delivery to patients with chronic pouchitis resulted in variable changes in microbiota composition, which were insufficient to impact on the microbiota function, mucosal immune responses or lead to clinical response for patients. This study demonstrates the necessity of functional as well as phylogenetic analysis of the microbiota following FMT.

This also raises several questions regarding FMT in IBD. What is the most appropriate protocol regarding route of administration, frequency of infusions and pre-treatment interventions? Which donors are most appropriate microbiologically? In which patient group will FMT be most effective - acute or chronic active disease, once remission has been induced by standard therapies or as a prophylactic approach? Is engraftment of particular species or a complex community that alters microbiota diversity necessary for effective functional change of the microbiota that has immunological and clinical sequelae? Further studies investigating the functionality of the microbiota post FMT in conjunction with immunological assessment post FMT are required to address these issues.

## Methods

The methods were carried out in accordance with ethical approval from local ethics committee (North London REC 2 EC no. 11/LO/0170) and all patients and donors gave their written informed consent. All experimental protocols were approved by NHS Research and Development at North West London Hospitals NHS Trust.

We conducted a pilot study of FMT in patients with chronic pouchitis diagnosed clinically, endoscopically and histologically, with a current pouch disease activity index (PDAI) ≥7^54^. Healthy donors included relatives, partners or anonymous unrelated donors. A single nasogastric infusion of donor faeces was given according to previously described protocols^48^,^49^ (Supplementary Methods). Briefly, donor stool was provided within six hours prior to faecal transplantation. 30 g of stool was homogenised with 50 ml of 0.9% saline to produce a faecal-saline solution and 30 ml of the faecal-saline solution was administered via the nasogastric tube.

Assessment before FMT consisted of clinical (PDAI and Cleveland global quality of life scores), endoscopic and histological assessment. Stool was also collected for analysis of faecal coliform sensitivities as previously described^55^. Stool and biopsies were collected for microbiological and metabonomic assessments and biopsies were collected for immunological assessments. These assessments were repeated four weeks following FMT (Supplementary Methods).

### Microbiota analysis

DNA was extracted, 16S rRNA gene PCR amplicons generated, and 454 pyrosequencing carried out as described previously^56^, except that Q5^TM^ *Taq* polymerase (New England Biolabs) was used for PCR reactions and 25 PCR cycles were used. “Blank” DNA extractions were processed concurrently, and were sequenced to monitor background contaminating bacterial species that were present in kits/laboratory reagents. All identified contaminant OTUs (Supplementary Table 3) were removed prior to further analyses.

Amplicons from each sample were pooled into an equimolar mix and then sequenced with the Lib-L kit using the 454 GS FLX Titanium platform. The 454 pyrosequence data has been deposited at the European Nucleotide Archive under Study Accession Number ERP005254/ Sample Accession Number ERS421606. The Golay barcode sequences used for each sample in the study are listed in the Supplementary Methods. 16S rRNA gene sequences were analysed, and Non-metric multidimensional scaling (NMDS) plot, dendrograms and AMOVA values calculated using the mothur software package^57^. OTUs and higher taxa that were differentially abundant between cohorts were assessed by Metastats^58^, as applied in mothur. Significance threshold levels were adjusted to mitigate against false discovery rate using the Benjamini-Hochberg method^59^ (Supplementary Methods).

### ^1^H NMR spectroscopy

^1^H NMR spectra of both fresh faecal water and dried faecal samples obtained from donors and patients at pre- and post-FMT were acquired using a Bruker 600 MHz spectrometer (Bruker, Rheinstetten, Germany) at the operating ^1^H frequency of 600.13 MHz at a temperature of 300 K. A standard NMR pulse sequence (recycle delay-90°-t_1_-90°-t_m_-90°-acquisition) was used to obtain standard one-dimensional ^1^H NMR spectral data, where t_1_ was set to 3 ms and t_m_ (mixing time) was set to 100 ms. The water peak suppression was achieved using selective irradiation during a recycle delay of 2 s and t_m_. A 90 degree pulse was adjusted to 10 μs. A total of 128 scans were collected into 64 k data points with a spectral width of 20 ppm (Supplementary Methods).

^1^H NMR spectra of faecal water samples were automatically phased, referenced to TSP at d^1^H 0.00 and baseline-corrected using an in-house developed MATLAB script (Supplementary Methods). Probabilistic quotient normalization was subsequently performed on the datasets in order to account for dilution of complex biological mixtures. Principal component analysis (PCA) and orthogonal partial least squares discriminant analysis (OPLS-DA) were carried out in SIMCA (P + 13.0) and MATLAB software in order to gain an overview of the variation in the spectral dataset and classification-related (e.g. donors vs. patients, pre-FMT vs. post-FMT) metabolic changes respectively.

OPLS regression analysis was used to correlate metabolic data with microbial profiles. Fifteen groups of bacteria at the family levels, each present in greater than 10 samples, were selected for correlation analysis (*Lachnospiraceae, Bacteroidaceae, Enterobacteriaceae, Prevotellaceae, Ruminococcaceae, Clostridiaceae, Lactobacillaceae, Peptostreptococcaceae, Sutterellaceae, Erysipelotrichaceae, Porphyromonadaceae, Rikenellaceae, Streptococcaceae, Pasteurellaceae, Veillonellaceae)* as well as unclassified *Clostridiales*, and then all remaining unclassified *Firmicutes*.

### Isolation of lamina propria dendritic cells and cell surface labelling

The method used has been described and validated previously^29^,^60^,^61^. Cells were labelled in FACS buffer (phosphate-buffered saline containing 1 mmol/L EDTA and 0.02% sodium azide). To prevent non-specific binding, unoccupied binding sites were blocked with foetal calf serum prior to antibodies being added at predetermined optimal concentrations (Supplementary Methods).

### Cytokines in whole biopsy culture supernatants

Cell-free culture supernatants were analysed using a multiplex assay (BD Cytometric Bead Array) according to the manufacturer’s instructions to determine levels of IL-6 and TNF in biopsy supernatants.

### Flow Cytometry

Data were acquired using a FACSCanto II flow cytometer (BD Biosciences, Oxford, England). Isotype matched controls were used for all markers assessed using multicolour analysis DCs were identified as an HLA-DR^+^ lineage^–^ (CD3^–^, CD14^–^,CD16^–^,CD19^–^,CD34^–^) population. Surface markers (TLR 2, 4 and 5, CCR 9, integrin β7 and CD40) were assessed using flow cytometric analysis. Super-enhanced Normalised Subtraction WinList version 5 software (Verity Software House, Maine) was used to measure the percentage of positive staining cells and the level of staining for cell surface markers as previously described^29^,^62^,^63^.

### Statistical Analysis

Clinical data are presented as median with range. Dendritic cell surface marker and cytokine expression data are shown as mean ± SEM. Wilcoxon signed rank test was used to compare clinical and immunological data pre- and post- faecal transplantation.

## Additional Information

**How to cite this article**: Landy, J. *et al*. Variable alterations of the microbiota, without metabolic or immunological change, following faecal microbiota transplantation in patients with chronic pouchitis. *Sci. Rep*. **5**, 12955; doi: 10.1038/srep12955 (2015).

## Supplementary Material

Supplementary Information

## Figures and Tables

**Table 1:** Patient characteristics.

Patient	Sex	Time sinceRPC (years)	Pouchtype	Pre-pouchileitis	ESBLcoliforms	Ciprofloxacinsensitive coliforms
1	F	6	J	Yes	No	Yes
2	F	6	J	Yes	No	No
3	F	10	J	Yes	Yes	No
4	F	22	W	Yes	No	Yes
5	M	16	J	Yes	Yes	No
6	M	22	W	Yes	Yes	No
7	M	4	J	Yes	No	Yes
8	F	4	J	Yes	No	Yes
