# Supplementary material for: Variable alterations of the microbiota, without metabolic or immunological change, following faecal microbiota transplantation in patients with chronic pouchitis
Source: Sci Rep. 2015 Aug 12;5:12955. doi: 10.1038/srep12955 (PMC4532993; doi:10.1038/srep12955)
Supplement: Supplementary Information [file srep12955-s1.pdf]

Supplementary Information

Variable alterations of the microbiota, without metabolic or immunological change, following faecal microbiota transplantation in patients with chronic pouchitis.

Landy J[1,2], Walker AW[3,4], Li JV[5], Al-Hassi HO[2], Ronde E[2], English NR[2], Mann ER[2], Bernardo D[2], McLaughlin S[6], Parkhill J[3], Ciclitira PJ[7], Clark SK[8], Knight SC[2], Hart AL [1,2]

1. **IBD Unit, Gastroenterology Dept. St Mark's Hospital, Harrow, London, UK**
2. **Antigen Presentation Research Group, Faculty of Medicine, Imperial College London, Northwick Park and St Mark's Campus, Harrow, UK**
3. **Pathogen Genomics Group, Wellcome Trust Sanger Institute, Hinxton, Cambridgeshire, UK**
4. **Microbiology Group, Rowett Institute of Nutrition and Health, University of Aberdeen, Greenburn Road, Aberdeen, UK**
5. **Centre for Digestive and Gut Health & Division of Computational and Systems Medicine, Department of Surgery and Cancer, Faculty of Medicine, Imperial College London, London, UK**
6. **School of Health and Social Care, Bournemouth University, Bournemouth, UK**
7. **Department of Gastroenterology, The Rayne Institute, St Thomas' Hospital, London, UK**
8. **Department of Surgery, St Mark's Hospital, Harrow, London, UK**

Supplementary Methods

Patient and donor identification

Patients were recruited from an adult tertiary pouch clinic at St Mark's Hospital, United Kingdom between July 2011 and July 2012. Chronic pouchitis was defined as confirmed pouchitis (diagnosed clinically, endoscopically and histologically; with a current pouch disease activity index (PDAI) ≥7) of more than four weeks duration not responsive to one or more antibiotics. Chronic antibiotic dependent pouchitis was defined as symptoms only controlled while maintained on antibiotics. Chronic antibiotic refractory pouchitis was defined as pouchitis that no longer responded to a single antibiotic [1].

FMT was offered to patients who were antibiotic dependent or refractory and had failed to respond to or had declined immunosuppressive therapies and/or defunctioning ileostomy. Stool samples were taken prior to study entry to exclude other pathogens including *Clostridium difficile*. Patients were excluded if they had used antibiotics or non-steroidal medications within 2 weeks of study participation.

Donors were screened by clinical questionnaire, serology and stool tests as per previous studies of FMT [2-5]. Healthy donors included relatives, partners or an anonymous unrelated donor. Healthy donors were excluded if they had a history of gastrointestinal illness, inflammatory bowel disease, bowel cancer, antibiotic or probiotic use within the preceding 6 months, or hospitalisation within 3 months of study participation. Screening of healthy donors included blood tests for full blood count, renal and liver function, serology for hepatitis A,B,C, E, CMV,

EBV, HTLV I/II, HIV and *Treponema pallidum*. Three stool samples were taken to exclude *Cryptosporidium*, *Salmonella* spp., *Shigella* spp., *Escherichia coli*, *Campylobacter jejuni*, *C. difficile* toxin, helminths, ova and parasites.

FMT Protocol

A single nasogastric infusion of donor faeces was given according to previously described protocols [2,3]. Stool donors were asked to provide a stool sample less than 6 hours prior to faecal transplantation. 30 g of stool was homogenised with a household blender in 50 ml of 0.9% saline until reaching a smooth consistency and filtered through sterile gauze to produce a faecal-saline solution. The night before and the morning of the procedure the recipient was treated with a proton pump inhibitor. A nasogastric tube was inserted and the position confirmed with chest radiograph and 30 ml of the faecal-saline solution was administered via the nasogastric tube which was then flushed with 50 ml of normal saline solution.

Assessment and sampling

Clinical assessment was made between zero and seven days prior to FMT. Pre-FMT assessment consisted of clinical (PDAI and Cleveland global quality of life scores) and endoscopic assessment and biopsies were taken for histological assessment. Stool was also collected for analysis of faecal coliform sensitivities as previously described [6].

Clinical end points were defined as the number of patients in clinical remission (Clinical PDAI =0/ total PDAI ≤4) or clinical response (reduction in PDAI score ≥3 points) [7] four weeks after treatment with FMT and the number of patients demonstrating changes in pouch faecal bacterial sensitivities following FMT.

Biopsies (n=2) for microbiological assessment were immediately washed in phosphate buffered saline (PBS) and snap frozen in liquid nitrogen and stored at -80◦C. 2 ml of stool for microbiological and metabolic assessment was mixed with 8 ml of PBS, vortexed and centrifuged at 250 g for 1 minute to exclude large particulate matter. Aliquots (1:10) were stored in RNA later (Ambion) at -80◦C. Biopsies were collected for isolation of dendritic cells (n=6) and for overnight culture (n=1) for assessment of cytokines in biopsy supernatants (see below).

Schedule of stool and mucosal biopsy analysis

0-7 days Pre-FMT analysis	4 weeks Post-FMT
Donor faecal bacterial DNA extracted – 16S rRNA gene amplicons generated	
Patient faecal bacterial DNA extracted – 16S rRNA gene amplicons generated	Patient faecal bacterial DNA extracted – 16S rRNA gene amplicons generated
Patient faecal metabonomic sample	Patient faecal metabonomic sample
Patient faecal coliform sensitivities	Patient faecal coliform sensitivities
Patient pouch mucosal bacterial DNA (samples taken at pouchoscopy 1)	Patient pouch mucosal bacterial DNA (samples taken at pouchoscopy 2)
Immunological analysis of patient pouch mucosal samples (samples taken at pouchoscopy 1)	Immunological analysis of patient pouch mucosal samples (samples taken at pouchoscopy 2)

PCR and 16S rRNA gene sequencing and analysis

16S rRNA genes were amplified using Golay barcoded primers (Eurofins MWG Operon). Bacterial primers 454-338F (5'- CCTATCCCCTGTGTGCCTTGGCAGTCT CAGACTCCTACGGGAGGCAGCAG-3') and 454-926R (5'-CCATCTCATCCCTGCGTGTCTCCGACTCAG-barcode-CCGTCAATTCMTTTRAGT-3'), which span variable regions V3 to V5 of the 16S rRNA gene were used. Each sample in the study was amplified with a 926R primer that contained a unique barcode sequence. The Golay barcodes that were used are as follows:

Sample	Barcode
Donor1_Faeces	GAGTGGTAGAGA
Patient1_Faeces_Pre	GCATAGTAGCCG
Patient1_Faeces_Post	GCATATAGTCTC
Patient1_Biopsy_Pre	GATCTCATAGGC
Patient1_Biopsy_Post	GATCTTCAGTAC
Donor2_Faeces	GATACGTCCTGA
Patient2_Faeces_Pre	GCATCGTCAACA
Patient2_Faeces_Post	GCATGTGCATGT
Patient2_Biopsy_Pre	GATGATCGCCGA
Patient2_Biopsy_Post	GATGCATGACGC
Donor3_Faeces	GATAGCTGTCTT
Patient3_Faeces_Pre	GCATTGCGTGAG
Patient3_Faeces_Post	GCCACTGATAGT
Patient3_Biopsy_Pre	GATGTCGTGTCA
Patient3_Biopsy_Post	GATGTGAGCGCT
Donor4_Faeces	GATAGTGCCACT
Patient4_Faeces_Pre	GCCAGAGTCGTA
Patient4_Faeces_Post	GCCTATACTACA

Patient4_Biopsy_Pre	GATTAGCACTCT
Patient4_Biopsy_Post	GCAATAGCTGCT
Donor5_Faeces	GATATGCGGCTG
Patient5_Faeces_Pre	GCGACTTGTGTA
Patient5_Faeces_Post	GCGAGATCCAGT
Patient5_Biopsy_Pre	GCACATCGAGCA
Patient5_Biopsy_Post	GCACGACAACAC
Donor6_Faeces	GATCAGAAGATG
Patient6_Faeces_Pre	GCGATATATCGC
Patient6_Faeces_Post	GCGGATGTGACT
Patient6_Biopsy_Pre	GCACTCGTTAGA
Patient6_Biopsy_Post	GCACTGAGACGT
Donor7_Faeces	GATCCGACACTA
Patient7_Faeces_Pre	GCGTACAACTGT
Patient7_Faeces_Post	GCGTATCTTGAT
Patient7_Biopsy_Pre	GCAGCACGTTGA
Patient7_Biopsy_Post	GCAGCCGAGTAT
Donor8_Faeces	GATCGCAGGTGT
Patient8_Faeces_Pre	GCGTTACACACA
Patient8_Faeces_Post	GCTAAGAGAGTA
Patient8_Biopsy_Pre	GCAGGATAGATA
Patient8_Biopsy_Post	GCAGGCAGTACT
Control1_Neg	GATCTATCCGAG
Control1_Pos	GATCGTCCAGAT
Control2_Neg	GCAGTTCATATC
Control3_Neg	GCTAGTCTGAAC
Control4_Neg	GGTGCGTGTATG
Control5_Neg	GTAGAGCTGTTC

Q5™ Taq polymerase (New England Biolabs) was used for PCR reactions according to the product protocol with the following PCR cycling conditions: 98 ◦C for 2mins, followed by 25 cycles of 98 ◦C for 30 secs, 52 ◦C for 30 secs and 72 ◦C for 2 mins, followed by a final extension of 72 ◦C for 5 mins.

16S rRNA gene amplicons from each sample were then pooled in equimolar amounts into a mastermix for sequencing using the Lib-L kit on the 454 GS FLX Titanium platform. The resulting sequence data was processed using the mothur software package [8] as described previously [9], except that sequences with less than a minimum length of 320 bp rather than 350 bp were discarded.

Any contaminant OTUs that were detected in the sequenced negative control samples were removed from the final dataset (see Supplementary Table 3 for full list of contaminants). Diversity comparisons (Chao, Shannon and inverse Simpson) were carried out after first sub-sampling the data down to 391 reads per sample to ensure equal sampling depth across all samples. Good's coverage (an estimate of completeness of species sampling) at 391 reads per sample was on average greater than 92% for all sample groups (overall median 95.9%, range 87.9 to 99.7%). Similarity indices were assessed using Bray-Curtis and Theta Yue & Clayton calculators using mothur. In order to identify differentially abundant taxa between study cohorts, the 50 most abundant taxa from the OTU, Genus and Family taxonomic levels, and all phyla, were assessed by Metastats [10], as applied in mothur. Significance thresholds were adjusted to account for false discovery rate when making multiple comparisons using the Benjamini-Hochberg approach [11].

Sample preparation for NMR spectroscopic analysis

A total of 800 µl of faecal slurry was spun for 10 min 10,000 *x g* and 540 µl of supernatant was taken to thoroughly mix with 60 µl of 1.5 M potassium phosphate buffer. The mixture was subsequently centrifuged again at 10,000 *x g* for 10 min and 550 µl of supernatant was transferred into an NMR tube with an outer diameter of 5 mm. Ethanol signals were observed in the NMR spectra of fresh faecal samples, some of which contained extremely high concentrations, which affect the spectral quality and subsequent data analysis. To remove ethanol, all faecal water samples were dried out using a speed vacuum, re-suspended in 650 µl D_2O and sonicated for 20 min. The resulting samples were spun for 5 min at 10,000 *x g* and 600 µl of supernatant was transferred into an NMR tube with an outer diameter of 5 mm. The water peak region [d^1H 4.7-4.92] were removed to minimise the effect of the disordered baseline caused by water suppression. For the dataset of fresh faecal samples, ethanol peaks were also removed. Probabilistic quotient normalization was subsequently performed on the datasets in order to account for dilution of complex biological mixtures.

Isolation of lamina propria dendritic cells

The method used has been described and validated previously [12-14]. The epithelium was removed after a 60 minute treatment with 1 mM EDTA in calcium- and magnesium-free Hank's balanced salt solution at 37°C with gentle agitation. The remaining biopsy tissue was then digested in 1mg/mL collagenase D (Roche Diagnostics Ltd, Lewes, England) in RPMI 1640/HEPES (Sigma-Aldrich Co Ltd, Poole, England) containing 2% foetal calf serum and

20 µg/mL deoxyribonuclease I (Roche Diagnostics Ltd) agitating for 90–120 minutes at 37°C. After incubation, lamina propria mononuclear cells (LPMC) released from the tissue samples were passed through a cell strainer and washed in complete medium.

Cell Surface Labelling

Cells were labelled in FACS buffer (phosphate-buffered saline containing 1mmol/L EDTA and 0.02% sodium azide). To prevent non-specific binding, unoccupied binding sites were blocked with foetal calf serum prior to antibodies being added at predetermined optimal concentrations. Following labelling, cells were washed twice in FACS buffer and resuspended in 300 µl 1% paraformaldehyde and stored at 4◦C until acquisition within 24 hours.

Antibodies

Antibodies with the following specificities and conjugations were used: β7 integrin- PE (FIB504), CD3-PeCy5 (UCHT1), CD16-PeCy5 (3G8), CD34-PeCy5 (581), HLA-DR-APC (G46-6) were purchased from BD Pharmingen. TLR 4-FITC (HTA125), TLR 2-FITC (TLR2.3),CD40-FITC (LOB7/6), CD14-PeCy5 (61D3),CD19-PeCy5 (H1B19) were from AbdSerotec. CCR 9-PE (112509) was purchased from R&D systems. TLR 5-FITC (85B152.5) was purchased from Abcam. Appropriate isotype-matched control antibodies were purchased from the same manufacturers.

Cytokines in whole biopsy culture supernatants

Biopsy samples were blotted and weighed prior to being cultured overnight in complete medium (RPMI 1640 Dutch modification (Sigma Aldrich Co. Ltd, Irvine, UK) supplemented with 2 mM L-glutamine, 100 $\mu g \cdot ml^{-1}$ streptomycin, 100 $units \cdot ml^{-1}$ penicillin and gentamicin (50 $\mu g \cdot ml^{-1}$) with 10% foetal calf serum) at 37◦C in a humidified 5% CO_2 atmosphere. Cell-free culture supernatants were analysed using a multiplex assay (BD Cytometric Bead Array) according to the manufacturer's instructions to determine levels of IL-6 and TNF in biopsy supernatants. Standard curves were plotted to calculate the detection limit of each cytokine. Values below the detection limit are reported as being equal to that level.

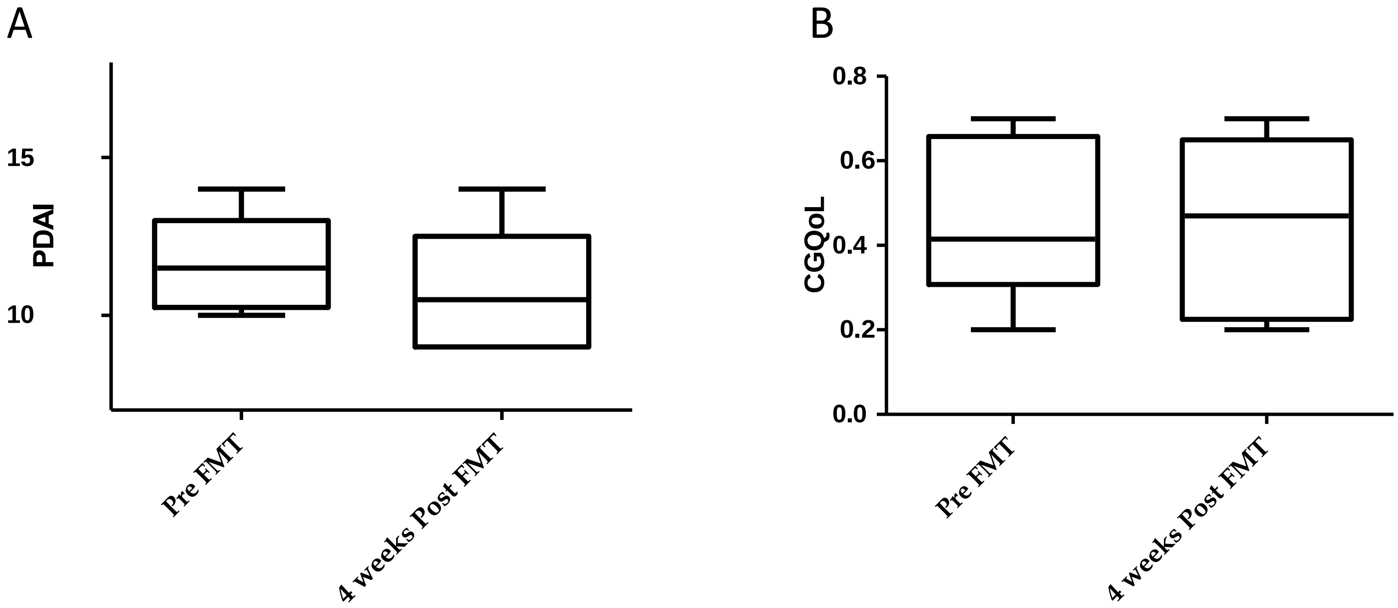

Supplementary figure 1. Clinical assessments pre and post FMT. A. Median total PDAI and B. Median Cleveland Global Quality of Life (CGQoL) score before and 4 weeks after FMT (n=8). There were no significant changes in either PDAI (11.5 range 10-14 pre and 10.5 range 9-14 post; ns) or CGQoL (0.41 range 0.2-0.7 pre and 0.47 range 0.2-0.7 post; ns) 4 weeks after FMT.

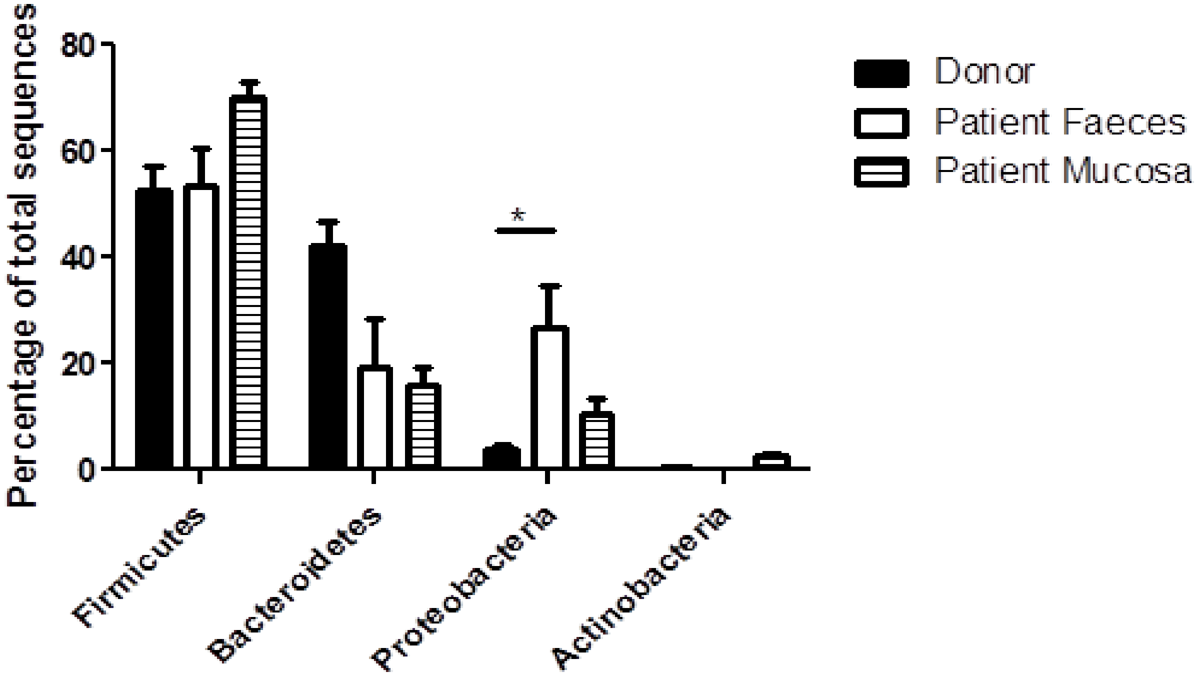

Supplementary figure 2. Percentage of sequences identified from the four predominant bacterial phyla in donor and patient samples at baseline. Percentage of total sequences from the predominant bacterial phyla in donor stool (n=8), patient stool pre FMT (n=7) and patient mucosal samples pre FMT (n=8).

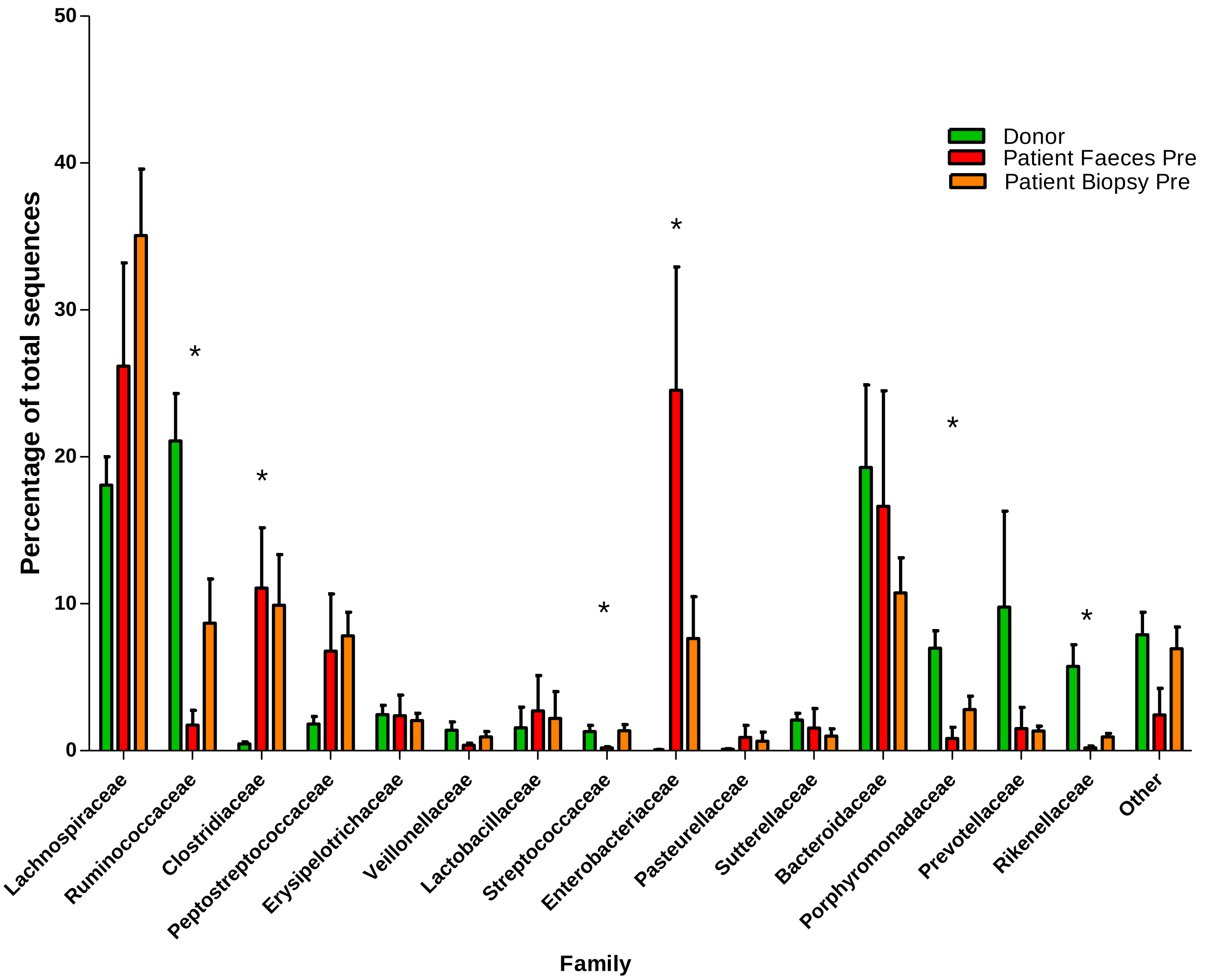

Percentage of total sequences
0
10
20
30
40
50
Donor
Patient Faeces Pre
Patient Biopsy Pre
Lachnospiraceae
Ruminococcaceae
Clostridiaceae
Peptostreptococcaceae
Erysipelotrichaceae
Veillonellaceae
Lactobacillaceae
Streptococcaceae
Enterobacteriaceae
Pasteurellaceae
Sutterellaceae
Bacteroidaceae
Porphyromonadaceae
Prevotellaceae
Rikenellaceae
Other
Family

Supplementary figure 3. Percentage of sequences identified from the bacterial families of >1% total abundance in donor and patient samples at baseline. Percentage of total sequences from the predominant bacterial families in donor stool (n=8), patient stool pre FMT (n=7) and patient mucosal samples pre FMT (n=8). * denote where differences between donor and patient stool are significant after adjustment for FDR.

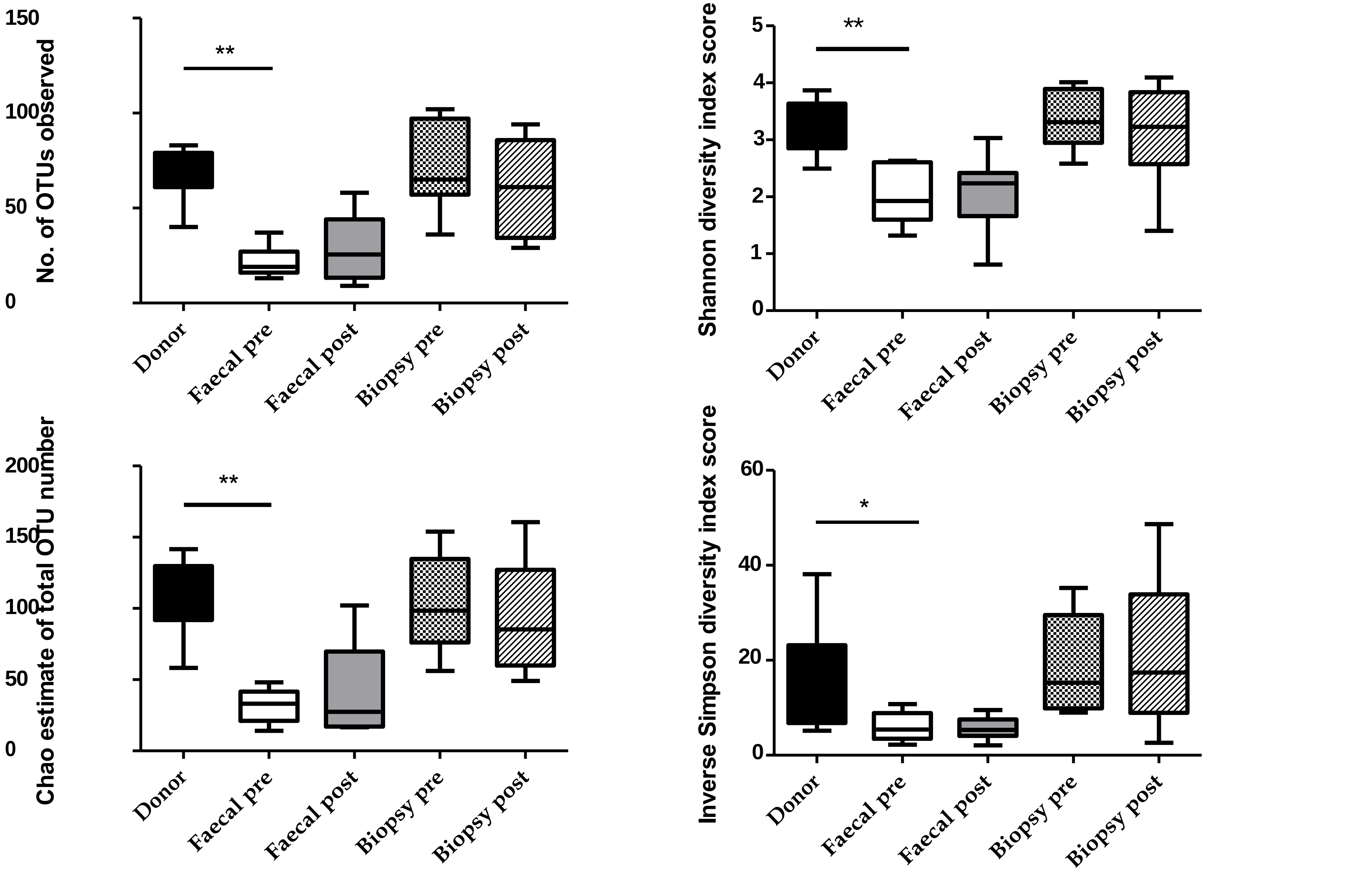

Supplementary figure 4. Diversity of donor and patient samples at baseline and post FMT. A. Comparison of the number of operational taxonomic units (OTUs) observed. B. Chao estimate of total OTU number. C. Shannon diversity index. D. Simpson Diversity index. donor stool (n=8), patient stool pre FMT (n=7), patient mucosal samples pre FMT (n=8), patient stool post FMT (n=8), patient mucosal samples post FMT (n=5). * and ** denote p values ≤0.05 and 0.01 respectively.

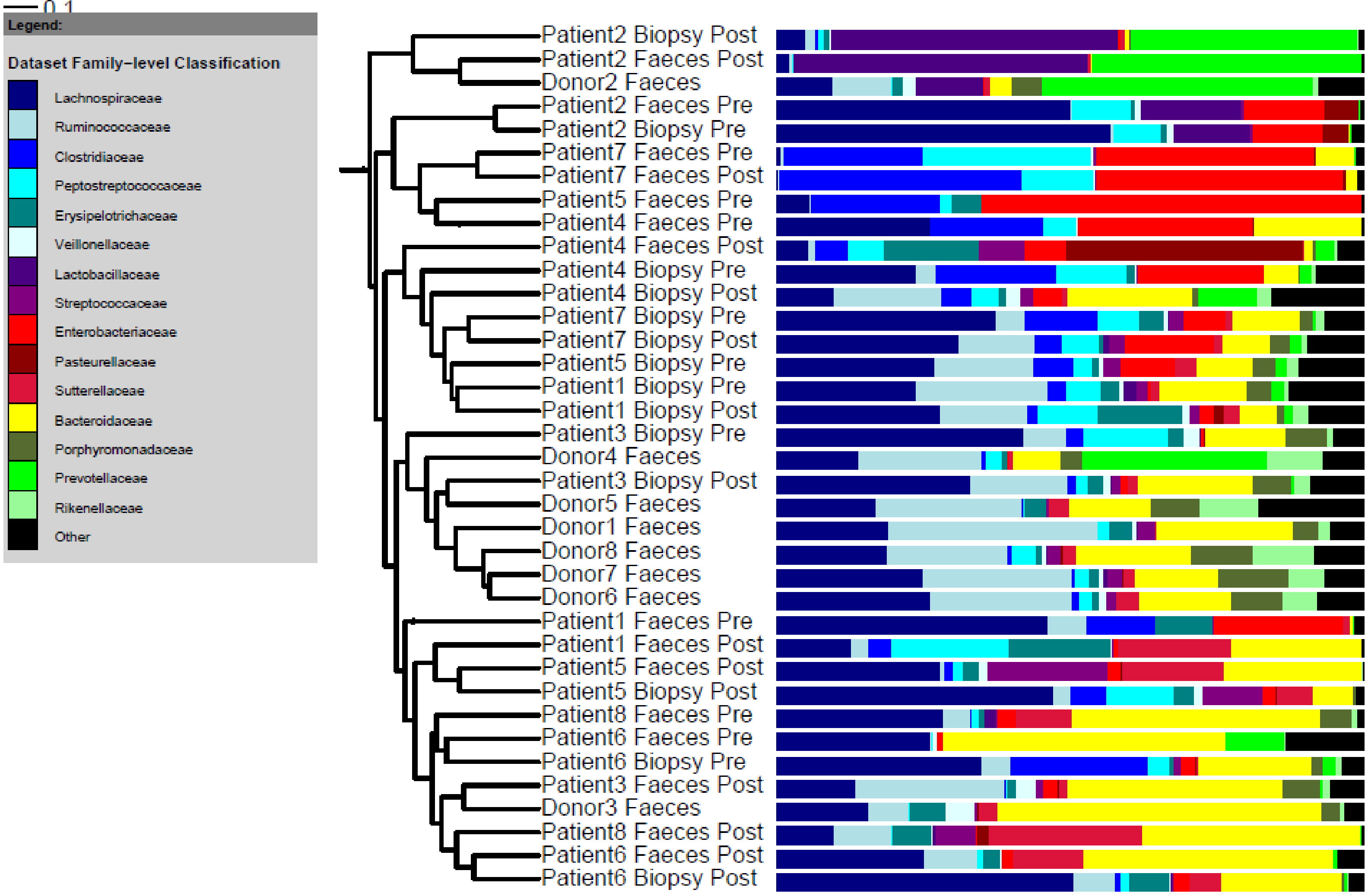

Supplementary figure 5. Bray Curtis dendrogram showing overall comparison between samples. This illustrates how some samples (e.g. from Patient 2) come to resemble the donor's microbiota following FMT, while others (e.g. Patient 7) remain distinct from the donor's microbiota following FMT.

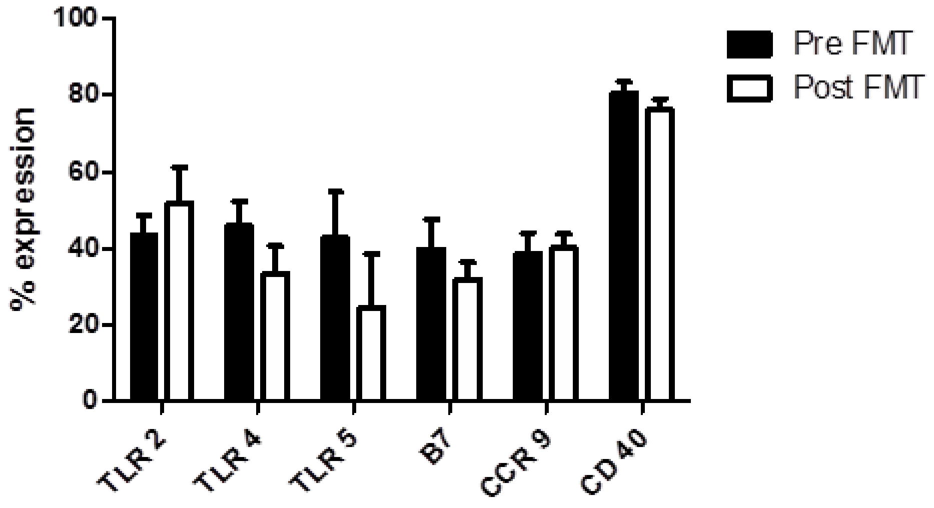

Supplementary figure 6. TLR 2, TLR 4, TLR 5 and CD40 and homing marker (β7 and CCR9) expression on HLA-DR+ lineage negative cells in pouch tissue pre and post FMT.

Genus	Donor stool (%)	Patient stool baseline (%)	p-value	Benjamini & Hochberg-adjusted Significance level
Bacteroides	19.3 +- 5.6	16.6 +- 7.8	0.855506	0.05
Escherichia_Shigella	0.06 +- 0.02	22.0 +-8.3	0.003545**	0.013
Prevotella	9.3 +-6.3	0.07 +-0.03	0.130065	0.029
Lachnospiracea_incertae_sedis	4.6+-0.6	10.6 +- 3.5	0.079571	0.026
Clostridium_sensu_stricto	0.45 +-0.1	11.1 +-4.1	0.007156**	0.016
Lactobacillus	1.6 +- 1.4	2.7 +- 2.4	0.783714	0.048
Faecalibacterium	10.9 +-2.1	1.4 +-0.9	0.000143**	0.004
Blautia	2.5 +-0.4	5.0 +-2.3	0.272416	0.036
Clostridium_XI	1.8 +-0.5	6.8 +-3.9	0.201351	0.034
unclassified Lachnospiraceae	4.6 +-0.8	2.8 +-1.0	0.154104	0.032
Sutterella	2.0 +-0.5	0.2 +-0.2	0.000623**	0.009
Anaerostipes	2.6 +-0.3	3.6 +-2.5	0.784571	0.049
Clostridium_XVIII	1.5 +-0.4	2.2 +-1.4	0.727416	0.046
Alistipes	5.7 +-1.5	0.2 +-0.1	0.000403**	0.006
Streptococcus	1.3 +-0.4	0.2 +-0.1	0.007506**	0.017
Clostridium_XlVa	0.9 +-0.4	1.4 +-0.9	0.730506	0.047
Barnesiella	2.8 +-0.5	0.1 +-0.1	0.000013**	0.002
Subdoligranulum	3.0 +-0.7	0.04 +-0.0	0.000455**	0.007
Roseburia	1.0 +-0.2	2.4+-2.3	0.68513	0.044

Supplementary table 1. Analysis of significant OTUs and genera from donor and patient samples at baseline

Genus	Patient stool baseline (%)	Patient stool post FMT (%)	p-value	Benjamini & Hochberg-adjusted Significance level	Patient mucosa baseline (%)	Patient mucosa post FMT (%)	p-value	Benjamini & Hochberg-adjusted Significance level
Bacteroides	16.6 +- 7.8	20.7 +-6.2	0.852183	0.044	10.7 +-2.4	11.9 +-3.2	0.681319	0.034
Escherichia_Shigella	22.0 +-8.3	4.5 +-2.6	0.01	0.003	7.3 +-2.8	3.2 +-1.1	0.256743	0.01
Prevotella	0.07 +-0.03	6.2 +-5.7	0.307067	0.015	0.93 +-0.3	7.5 +_5.4	0.350649	0.014
Lachnospiracea_incertae_sedis	10.6 +- 3.5	4.0 +-1.4	0.040517	0.004	11.7 +-3.6	9.2 +-2.5	0.619381	0.029
Clostridium_sensu_stricto	11.1 +-4.1	6.5 +-5.0	0.73575	0.037	9.9 +-3.5	2.9 +-0.8	0.104895	0.004
Lactobacillus	2.7 +- 2.4	6.4 +-6.2	0.803133	0.04	1.9 +- 1.6	7.2 +-7.0	0.812188	0.044
Faecalibacterium	1.4 +-0.9	5.2 +-2.4	0.10195	0.007	4.6 +-1.7	6.1 +-1.3	0.518482	0.024
Blautia	5.0 +-2.3	2.3 +-0.8	0.241933	0.013	7.2 +- 2.3	5.0 +-1.6	0.476523	0.02
Clostridium_XI	6.8 +-3.9	5.2 +-2.6	0.877333	0.045	7.8 +-1.6	5.2 +-1.6	0.493506	0.022
unclassified Lachnospiraceae	2.8 +-1.0	2.0 +-0.6	0.75135	0.039	6.7 +-0.9	5.6 +-1.5	0.679321	0.033
Sutterella	0.2 +-0.2	9.7 +-3.7	0.004233	0.001	1.0 +-0.5	2.7 +-0.8	0.228771	0.009
Anaerostipes	3.6 +-2.5	1.4 +-0.7	0.6431	0.032	2.7 +-0.7	3.6 +-1.2	0.602398	0.028
Clostridium_XVIII	2.2 +-1.4	3.4 +-1.7	0.809817	0.041	1.2 +-0.4	3.5 +-1.8	0.085914	0.003
Alistipes	0.2 +-0.1	0.2+-0.2	0.9211	0.046	0.9 +-0.2	1.3 +-0.5	0.653347	0.03
Streptococcus	0.2 +-0.1	4.6 +-2.6	0.045	0.005	1.3 +-0.4	2.6 +-1.3	0.21978	0.008
Clostridium_XlVa	1.4 +-0.9	1.4+-0.8	0.981317	0.049	1.8 +-0.5	2.9+-0.9	0.368631	0.017
Barnesiella	0.1 +-0.1	0.7+-0.	0.554633	0.024	1.1 +-0.7	1.2 +-0.6	0.686314	0.035
Subdoligranulum	0.04 +-0.0	0.5 +-0.3	0.113983	0.009	1.0 +-0.5	1.2 +-0.4	0.318681	0.012
Roseburia	2.4+-2.3	0.2 +-0.1	0.524033	0.021	2.7 +-2.0	0.8 +-0.2	0.668332	0.031

Supplementary table 2. Analysis of genera from patient stool and mucosal samples pre and post FMT

Genus	Family	Order	Class	Phylum
Ralstonia(100)	Burkholderiaceae(100)	Burkholderiales(100)	Betaproteobacteria(100)	Proteobacteria(100)
Methylobacterium(100)	Methylobacteriaceae(100)	Rhizobiales(100)	Alphaproteobacteria(100)	Proteobacteria(100)
Rhodococcus(100)	Nocardiaceae(100)	Actinomycetales(100)	Actinobacteria(100)	Actinobacteria(100)
Acinetobacter(100)	Moraxellaceae(100)	Pseudomonadales(100)	Gammaproteobacteria(100)	Proteobacteria(100)
Pseudomonas(100)	Pseudomonadaceae(100)	Pseudomonadales(100)	Gammaproteobacteria(100)	Proteobacteria(100)
Curvibacter(54)	Comamonadaceae(100)	Burkholderiales(100)	Betaproteobacteria(100)	Proteobacteria(100)
Bradyrhizobium(57)	Bradyrhizobiaceae(100)	Rhizobiales(100)	Alphaproteobacteria(100)	Proteobacteria(100)
Massilia(100)	Oxalobacteraceae(100)	Burkholderiales(100)	Betaproteobacteria(100)	Proteobacteria(100)
Alcaligenes(100)	Alcaligenaceae(100)	Burkholderiales(100)	Betaproteobacteria(100)	Proteobacteria(100)
Methylobacterium(100)	Methylobacteriaceae(100)	Rhizobiales(100)	Alphaproteobacteria(100)	Proteobacteria(100)
Microbacterium(100)	Microbacteriaceae(100)	Actinomycetales(100)	Actinobacteria(100)	Actinobacteria(100)
Aquabacterium(91)	Burkholderiales_incertae_sedis(100)	Burkholderiales(100)	Betaproteobacteria(100)	Proteobacteria(100)
Dyadobacter(100)	Cytophagaceae(100)	Sphingobacteriales(100)	Sphingobacteria(100)	Bacteroidetes(100)
Roseomonas(100)	Acetobacteraceae(100)	Rhodospirillales(100)	Alphaproteobacteria(100)	Proteobacteria(100)
Dyadobacter(100)	Cytophagaceae(100)	Sphingobacteriales(100)	Sphingobacteria(100)	Bacteroidetes(100)
Sphingomonas(100)	Sphingomonadaceae(100)	Sphingomonadales(100)	Alphaproteobacteria(100)	Proteobacteria(100)
Subtercola(58)	Microbacteriaceae(100)	Actinomycetales(100)	Actinobacteria(100)	Actinobacteria(100)
unclassified(100)	unclassified(100)	Rhodospirillales(100)	Alphaproteobacteria(100)	Proteobacteria(100)
Pseudoclavibacter(67)	Microbacteriaceae(100)	Actinomycetales(100)	Actinobacteria(100)	Actinobacteria(100)
Tsukamurella(100)	Tsukamurellaceae(100)	Actinomycetales(100)	Actinobacteria(100)	Actinobacteria(100)
Sphingopyxis(100)	Sphingomonadaceae(100)	Sphingomonadales(100)	Alphaproteobacteria(100)	Proteobacteria(100)
Novosphingobium(100)	Sphingomonadaceae(100)	Sphingomonadales(100)	Alphaproteobacteria(100)	Proteobacteria(100)
Pedobacter(100)	Sphingobacteriaceae(100)	Sphingobacteriales(100)	Sphingobacteria(100)	Bacteroidetes(100)
Aeromicrobium(100)	Nocardioidaceae(100)	Actinomycetales(100)	Actinobacteria(100)	Actinobacteria(100)
Flavobacterium(100)	Flavobacteriaceae(100)	Flavobacteriales(100)	Flavobacteria(100)	Bacteroidetes(100)
Luteimonas(75)	Xanthomonadaceae(100)	Xanthomonadales(100)	Gammaproteobacteria(100)	Proteobacteria(100)
Comamonas(100)	Comamonadaceae(100)	Burkholderiales(100)	Betaproteobacteria(100)	Proteobacteria(100)
Mycobacterium(100)	Mycobacteriaceae(100)	Actinomycetales(100)	Actinobacteria(100)	Actinobacteria(100)
Sphingobium(100)	Sphingomonadaceae(100)	Sphingomonadales(100)	Alphaproteobacteria(100)	Proteobacteria(100)
unclassified(100)	unclassified(100)	unclassified(100)	unclassified(100)	Proteobacteria(100)
Rhizobium(100)	Rhizobiaceae(100)	Rhizobiales(100)	Alphaproteobacteria(100)	Proteobacteria(100)
Novosphingobium(100)	Sphingomonadaceae(100)	Sphingomonadales(100)	Alphaproteobacteria(100)	Proteobacteria(100)
Aurantimonas(100)	Aurantimonadaceae(100)	Rhizobiales(100)	Alphaproteobacteria(100)	Proteobacteria(100)
Pedobacter(100)	Sphingobacteriaceae(100)	Sphingobacteriales(100)	Sphingobacteria(100)	Bacteroidetes(100)
Ochrobactrum(100)	Brucellaceae(100)	Rhizobiales(100)	Alphaproteobacteria(100)	Proteobacteria(100)
Geodermatophilus(100)	Geodermatophilaceae(100)	Actinomycetales(100)	Actinobacteria(100)	Actinobacteria(100)
Pseudomonas(100)	Pseudomonadaceae(100)	Pseudomonadales(100)	Gammaproteobacteria(100)	Proteobacteria(100)
Paenibacillus(100)	Paenibacillaceae_1(100)	Bacillales(100)	Bacilli(100)	Firmicutes(100)
Pedobacter(100)	Sphingobacteriaceae(100)	Sphingobacteriales(100)	Sphingobacteria(100)	Bacteroidetes(100)
unclassified	Microbacteriaceae(100)	Actinomycetales(100)	Actinobacteria(100)	Actinobacteria(100)
unclassified(100)	Oxalobacteraceae(100)	Burkholderiales(100)	Betaproteobacteria(100)	Proteobacteria(100)
Sphingomonas(100)	Sphingomonadaceae(100)	Sphingomonadales(100)	Alphaproteobacteria(100)	Proteobacteria(100)
unclassified(100)	Bradyrhizobiaceae(100)	Rhizobiales(100)	Alphaproteobacteria(100)	Proteobacteria(100)
Chryseobacterium(100)	Flavobacteriaceae(100)	Flavobacteriales(100)	Flavobacteria(100)	Bacteroidetes(100)
Patulibacter(100)	Patulibacteraceae(100)	Solirubrobacterales(100)	Actinobacteria(100)	Actinobacteria(100)
Pseudomonas(100)	Pseudomonadaceae(100)	Pseudomonadales(100)	Gammaproteobacteria(100)	Proteobacteria(100)
unclassified(100)	unclassified(100)	Myxococcales(100)	Deltaproteobacteria(100)	Proteobacteria(100)
Sphingomonas(100)	Sphingomonadaceae(100)	Sphingomonadales(100)	Alphaproteobacteria(100)	Proteobacteria(100)
Methylophilus(100)	Methylophilaceae(100)	Methylophilales(100)	Betaproteobacteria(100)	Proteobacteria(100)
Methylobacterium(100)	Methylobacteriaceae(100)	Rhizobiales(100)	Alphaproteobacteria(100)	Proteobacteria(100)
unclassified(100)	Beutenbergiaceae(100)	Actinomycetales(100)	Actinobacteria(100)	Actinobacteria(100)
unclassified(100)	unclassified(100)	Sphingobacteriales(100)	Sphingobacteria(100)	Bacteroidetes(100)
unclassified(100)	Erythrobacteraceae(100)	Sphingomonadales(100)	Alphaproteobacteria(100)	Proteobacteria(100)
Dietzia(100)	Dietziaceae(100)	Actinomycetales(100)	Actinobacteria(100)	Actinobacteria(100)
Deinococcus(100)	Deinococcaceae(100)	Deinococcales(100)	Deinococci(100)	Deinococcus-Thermus(100)

Supplementary Table 3: Taxonomic list of contaminant OTUs that were removed from the final analysis after detecting their presence in negative "blank" control samples.

1 McLaughlin, S. D., Clark, S. K., Tekkis, P. P., Ciclitira, P. J. & Nicholls, R. J. Review article: restorative proctocolectomy, indications, management of complications and follow-up - a guide for gastroenterologists. *Alimentary Pharmacology & Therapeutics* 27, 895-909, doi:10.1111/j.1365-2036.2008.03643.x (2008).

2 Aas, J., Gessert, C. E. & Bakken, J. S. Recurrent Clostridium difficile colitis: case series involving 18 patients treated with donor stool administered via a nasogastric tube. *Clin Infect Dis* 36, 580-585, doi:10.1086/367657 (2003).

3 MacConnachie, A. A., Fox, R., Kennedy, D. R. & Seaton, R. A. Faecal transplant for recurrent Clostridium difficile-associated diarrhoea: a UK case series. *QJM* 102, 781-784, doi:10.1093/qjmed/hcp118 (2009).

4 Garborg, K., Waagsbo, B., Stallemo, A., Matre, J. & Sundoy, A. Results of faecal donor instillation therapy for recurrent Clostridium difficile-associated diarrhoea. *Scand J Infect Dis* 42, 857-861, doi:10.3109/00365548.2010.499541 (2010).

5 van Nood, E. *et al.* Duodenal Infusion of Donor Feces for RecurrentClostridium difficile. *New England Journal of Medicine*, 130116140046009, doi:10.1056/NEJMoa1205037 (2013).

6 McLaughlin, S. D. *et al.* Fecal Coliform Testing to Identify Effective Antibiotic Therapies for Patients With Antibiotic-Resistant Pouchitis. *Clinical Gastroenterology and Hepatology* 7, 545-548, doi:10.1016/j.cgh.2009.01.002 (2009).

7 Abdelrazeq, A. S., Kelly, S. M., Lund, J. N. & Leveson, S. H. Rifaximin-ciprofloxacin combination therapy is effective in chronic active refractory pouchitis. *Colorectal Dis* 7, 182-186, doi:10.1111/j.1463-1318.2004.00746.x (2005).

8 Schloss, P. D. *et al.* Introducing mothur: Open-Source, Platform-Independent, Community-Supported Software for Describing and Comparing Microbial Communities. *Applied and Environmental Microbiology* 75, 7537-7541, doi:10.1128/aem.01541-09 (2009).

9 Cooper, P. *et al.* Patent human infections with the whipworm, Trichuris trichiura, are not associated with alterations in the faecal microbiota. *PLoS One* 8, e76573, doi:10.1371/journal.pone.0076573 (2013).

10 White, J. R., Nagarajan, N. & Pop, M. Statistical methods for detecting differentially abundant features in clinical metagenomic samples. *PLoS Comput Biol* 5, e1000352, doi:10.1371/journal.pcbi.1000352 (2009).

11 Y Benjamini, Y. H. Y. Controlling the False Discovery Rate: A practical and powerful approach to multiple testing. . *Journal of the Royal Statistical Society* B57, 289-300 (1995).

12 Bell, S. J. *et al.* Migration and maturation of human colonic dendritic cells. *J Immunol* 166, 4958-4967 (2001).

13 Hart, A. *et al.* Characteristics of Intestinal Dendritic Cells in Inflammatory Bowel Diseases. *Gastroenterology* 129, 50-65, doi:10.1053/j.gastro.2005.05.013 (2005).

14 Al-Hassi, H. O. *et al.* A mechanistic role for leptin in human dendritic cell migration: differences between ileum and colon in health and Crohn’s disease. *Mucosal Immunology* 6, 751-761, doi:10.1038/mi.2012.113 (2012).
